# GLUT1 and GLUT3 involvement in anthocyanin gastric transport- Nanobased targeted approach

**DOI:** 10.1038/s41598-018-37283-2

**Published:** 2019-01-28

**Authors:** Hélder Oliveira, Catarina Roma-Rodrigues, Ana Santos, Bruno Veigas, Natércia Brás, Ana Faria, Conceição Calhau, Victor de Freitas, Pedro V. Baptista, Nuno Mateus, Alexandra R. Fernandes, Iva Fernandes

**Affiliations:** 1 REQUIMTE/LAQV, Department of Chemistry and Biochemistry, Faculty of Sciences, University of Porto, 4169-007 Porto, Portugal; 2 UCIBIO, Departamento de Ciências da Vida, Faculdade de Ciências e Tecnologia, Universidade Nova de Lisboa, Campus de Caparica, 2829-516 Caparica, Portugal; 3 REQUIMTE/UCIBIO, Department of Chemistry and Biochemistry, Faculty of Sciences, University of Porto, 4169-007 Porto, Portugal; 4 Nutrition & Metabolism, NOVA Medical School, Faculdade Ciências Médicas Universidade Nova de Lisboa, 1169-056 Lisboa, Portugal; 5 CINTESIS, Center for Health Technology and Services Research, Porto, Portugal; 6 Comprehensive Health Research Centre, NOVA Medical School, Faculdade Ciências Médicas, Universidade Nova de Lisboa, 1169-056 Lisboa, Portugal

## Abstract

Anthocyanins may protect against a myriad of human diseases. However few studies have been conducted to evaluate their bioavailability so their absorption mechanism remains unclear. This study aimed to evaluate the role of two glucose transporters (GLUT1 and GLUT3) in anthocyanins absorption in the human gastric epithelial cells (MKN-28) by using gold nanoparticles to silence these transporters. Anthocyanins were purified from purple fleshed sweet potatoes and grape skin. Silencing of *GLUT1* and/or *GLUT3* mRNA was performed by adding AuNP@GLUT1 and/or AuNP@GLUT3 to MKN-28 cells. Downregulation of mRNA expression occurred concomitantly with the reduction in protein expression. Malvidin-3-O-glucoside (Mv3glc) transport was reduced in the presence of either AuNP@GLUT1 and AuNP@GLUT3, and when both transporters were blocked simultaneously. Peonidin-3-(6′-hydroxybenzoyl)-sophoroside-5-glucoside (Pn3HBsoph5glc) and Peonidin-3-(6′-hydroxybenzoyl-6″-caffeoyl)-sophoroside-5-glucoside (Pn3HBCsoph5glc) were assayed to verify the effect of the sugar moiety esterification at glucose B in transporter binding. Both pigments were transported with a lower transport efficiency compared to Mv3glc, probably due to steric hindrance of the more complex structures. Interestingly, for Pn3HBCsoph5glc although the only free glucose is at C5 and the inhibitory effect of the nanoparticles was also observed, reinforcing the importance of glucose on the transport regardless of its position or substitution pattern. The results support the involvement of GLUT1 and GLUT3 in the gastric absorption of anthocyanins.

## Introduction

Positive correlations have been established between the consumption of flavonoid-rich foods and health benefits, in different *in vitro* and animal studies, but also in several epidemiological studies^1,2^. The beneficial effect of these foodstuffs has been attributed to the presence of polyphenolic compounds including anthocyanins. Although the consumption of anthocyanins may easily reach 200 mg/day, their bioavailability has been reported to be quite low (<1%)^3^. Anthocyanins are poorly absorbed as genuine parent glycosides or detected in blood as metabolites^4,5^.

The bioavailability of these compounds cannot be addressed only from a simple nutritional perspective. These pigments have unique physical-chemical properties that affect their behavior *in vivo*. The bioavailability of anthocyanins is the most difficult one to assess amongst all flavonoid compounds as a result of their occurrence under different structures in equilibrium depending on pH. In addition, biological samples are complex matrices making it hard to perform a clear chemical profile to properly identify and quantify bioactive molecules.

Anthocyanins are also very unstable at neutral pH and physiological temperature, and can interact with proteins or carbohydrates. Also, the bioavailable forms of anthocyanins *in vivo* are not exclusively the same that occur in food since they are also largely metabolized yielding several types of metabolites^4^. Considering *in vivo* conditions, anthocyanins are readily metabolized, degraded or excreted from the organism.

Due to their rapid appearance in plasma, the absorption of anthocyanins is also likely to occur at the gastric level, although the information on this topic is scarce^3^. Preliminary studies with a gastric cell barrier (MKN-28) model indicated that anthocyanins uptake involves a saturable transport but the absorption mechanism remains unknown^6^. Glucose transporters have been suggested as the main transporters involved in the absorption of these nutraceuticals^7^.

To further elucidate the role of glucose transporters in the uptake mechanism of anthocyanins in this gastric cell barrier model, a nano-based approach was explored herein using gold nanoparticles (AuNPs) functionalized with specific antisense hairpins for *GLUT1* and *GLUT3* gene silencing.

AuNPs, due to their extraordinary physical-chemical properties (i.e. high surface-to-volume ratio, allowing surface modification with a plethora of molecules for specific targeting and reduced size allowing interaction with biomolecules in a one-to-one scale), intrinsic chemical stability and apparent lack of toxicity, can be used as a vectorization tool to specifically and selectively silence gene expression, with greater efficiency over commercial available transfection agents like lipofectamine^8,9^. AuNPs have been used as vehicles to deliver silencing moieties (e.g. antisense oligonucleotides, siRNA) to silence genes involved in several cellular processes^10–15^.

Four anthocyanins, with a glucose moiety at different positions were assayed in this study: Malvidin-3-*O*-glucoside (Mv3glc), Peonidin-3-*O*-glucoside (Pn3glc), Peonidin-3-(6′-hydroxybenzoyl)-sophoroside-5-glucoside (Pn3HBsoph5glc) and Peonidin-3-(6′-hydroxybenzoyl-6″-caffeoyl)-sophoroside-5-glucoside (Pn3HBCsoph5glc). This work aims to apply novel and useful means yielding new data to comprehend how anthocyanins from different natural sources are absorbed using the *in vitro* human stomach cell model, MKN-28.

## Methods

### Purification of anthocyanin from red fruits and vegetables

Grape skin anthocyanins (*Vitis vinifera*) were extracted with an aqueous solution of methanol (1:1) acidified with HCl, for 2 days at room temperature. The *Vitis vinifera* grape anthocyanin extract was filtered in a 50 μm nylon membrane and then purified by TSK Toyopearl gel column (250 × 16 mm i.d.) chromatography according to the procedure described previously^16^. The extract was freeze-dried and stored at −18 °C until use.

Purple fleshed sweet potatoes (PFSP) were cut in slices and anthocyanins were extracted in 70% ethanol with ultra-sound assistance for 1 h. The obtained extract was centrifuged at 2,800 × *g* for 15 min to remove insoluble materials. The resulting supernatant was filtered and phenolic acids removed with Liquid-Liquid extraction (ethyl acetate/water, 1:1). The resulting extract was applied on a XAD-7HP column. Water was used to remove proteins, sugars and other interfering materials, and methanol used to recover anthocyanins. The enriched anthocyanin fraction was applied on a C-18 column to remove any remaining sugars. The extract was freeze-dried and stored at −18 °C until use.

Further HPLC preparative chromatography of the total anthocyanin extracts was performed to obtain purified Mv3glc, Pn3glc, Pn3HBsoph5glc and Pn3HBCsoph5glc. The purity and structural characterization of the three pigments was confirmed by HPLC-DAD-MS and NMR.

### HPLC analysis

HPLC analysis of anthocyanins was performed on Dionex Ultimate 3000 (Thermo Scientific; USA) equipped with a 250 × 4.6 mm i.d. reversed-phase C18 column (Merck, Darmstadt, Germany). Detection was carried out at 520 nm using a diode array detector (DAD). The solvents were (A) H_2_O/HCOOH (9:1) and (B) H_2_O/HCOOH/CH_3_CN (6:1:3). The gradient consisted of 20–52.5% B for 35 minutes at a flow rate of 1.0 mL/min. The column was washed with 100% B for 15 minutes and then stabilized at the initial conditions for another 15 minutes.

### Au-nanoconjugate Synthesis and Characterization

Synthesis of AuNPs with 14 nm of diameter was performed by the citrate reduction method^17^ and functionalization as previously described (Conde *et al*.^11^. AuNPs were functionalized with poly(ethylene glycol) (PEG) modified with a thiol group (AuNP@PEG) to obtain a 30% of coverage. AuNP@PEG were subsequently functionalized with antisense thiolated oligonucleotides for each target gene: *GLUT1*/*SLC2A1* 5′-GCTATGACATGAGGCGACCCGTCAGCTTCATAGC-3′ and *GLUT3*/*SLC2A3* 5′-TTTCGGATCTAATTCAAGTCTTCAAGCCGAAA-3′ (palindromic sequence underlined) (AuNP@PEG@anti-*GLUT1* and AuNP@PEG@anti-*GLUT3*). Au-nanoconjugates were prepared at a 1:100 ratio (AuNP:oligonucleotide) and centrifuged at 14,000 × *g* for 40 minutes, the precipitate was washed three times with RNAse free water. The number of oligonucleotides bound to the surface of AuNPs was determined using Quant-iT Oligreen ssDNA reagent (ThermoFisher Scientific). The Au-nanoconjugates were stored at 4 °C in the dark and characterized by UV-Vis spectroscopy, Transmission Electron Microscopy (TEM) and Dynamic Light Scattering (DLS).

### Cell culture

The MKN-28 cells originating from human gastric epithelium were kindly provided from IPATIMUP (Porto, Portugal). Cells were maintained in RPMI medium (RPMI 1640 (LifeTechnologies) supplemented with 10% (v/v) fetal bovine serum (FBS, LifeTechnologies), non-essential aminoacids (MEM, LifeTechnologies) and mixture of 100 U/mL Penicillin and 100 μg/mL Streptomycin (LifeTechnologies)), at 37 °C, 5% (v/v) of CO_2_ and 99% (v/v) of relative humidity.

### *GLUT1* and *GLUT3* silencing

MKN-28 cells (2 × 10^5^) were initially seeded on 35 mm disks, and incubated for 24 h at 37 °C, 5% (v/v) CO_2_ and 99% (v/v) relative humidity to obtain a cell monolayer with 75% confluence. Afterwards, the medium was replaced by fresh RPMI medium supplemented with AuNP@PEG@anti-*GLUT1* or AuNP@PEG@anti-*GLUT3* at an oligonucleotide concentration of 20 nM and 30 nM for 9 h, 12 h and 24 h. For control purposes, cells were challenged in parallel with 0.63 nM AuNP@PEG, corresponding to 20 nM of oligonucleotide (AuNPs:oligonucleotide ratio = 1:30), or 0.75 nM AuNP@PEG, corresponding to 30 nM of oligonucleotide (AuNPs:oligonucleotide ratio = 1:40).

After cell detachment using TrypleExpress (LifeTechnologies), cells were pelleted by centrifugation at 500 × *g* for 5 min at room temperature, and total RNA extracted using SV Total RNA Isolation System (Promega) according to the manufacture’s protocol. cDNA was synthetized from total RNA (100 ng) using NZY M-MuLV First-Strand cDNA Synthesis kit (NZYtech) according to manufacturer’s instructions.

Expression of *GLUT1* and *GLUT3* was evaluated using the Kapa SYBR Fast qPCR Master Mix (KAPABIOSYSTEMS) with the following primers at a final concentration of 0.08 µM each: *GLUT1* forward (5′-CAATGCTGATGATGAACCTG-3′) and *GLUT1* reverse (5′-GGGATGAAGATGATGCTCA-3′); *GLUT3* forward (5′–ATGGGGACACAGAAGGTCACC-3′) and *GLUT3* reverse (5′–AGCCACCAGTGACAGCCAAC-3′). The RT-qPCR was performed in a Corbett Rotor-Gene 6000 thermal cycler (QIAGEN) with the following conditions, initial denaturation at 95 °C for 5 min and 30 cycles of 95 °C for 45 seconds, 62 °C for 25 seconds and 72 °C for 45 seconds. Expression data were analyzed by the Ct method (2^−∆∆Ct^)^18^ using *GAPDH* as a housekeeping gene ^19^ and cells treated with AuNP@PEG as control. Three independent biological replicates were performed for each condition and a significative gene silencing result was considered when 2^−∆∆Ct^ < 0.5, and *p-value* < 0.05 (using t-test in GraphPad Prism vs 6.0 software).

### Simultaneous silencing of both transporters

After identifying the best conditions for the effective silencing of *GLUT1* or *GLUT3* (Supplementary Information), the most suitable strategy for silencing both genes under the conditions required for transport studies was evaluated. MKN-28 cells were seeded in 24 well plates with a density of 1 × 10^5^ cells/mL in RPMI medium. Cells were incubated for 5 days at 37 °C, 5% (v/v) CO_2_, 99% (v/v) relative humidity, and the medium changed by fresh medium every two days. After reach a uniform layer of cells covering the bottom of the well, cells were challenged with RPMI medium supplemented with 5.5 mM fructose (Merck KGaA, Darmstadt, Germany) and: a) 30 nM AuNP@PEG@anti-*GLUT1* (AuNP@GLUT1 condition), b) 20 nM AuNP@PEG@anti-*GLUT3* (AuNP@GLUT3 condition), c) 30 nM AuNP@PEG@anti-*GLUT1* + 20 nM AuNP@PEG@anti-*GLUT3* (AuNP@GLUT1 + 3 condition), d) 0.75 nM AuNP@PEG (AuNP@PEG1 condition, control of a)), e) 0.63 nM AuNP@PEG (AuNP@PEG3 condition, control of b)), or f) 1.38 nM AuNP@PEG (AuNP@PEG1 + 3 condition, control of c)). For control purposes, cells were also challenged with RPMI medium supplemented only with 5.5 mM fructose. After 24 h of incubation, cells were collected (24 h time point) or the medium replaced by fresh medium supplemented with the same concentration of nanoparticles as used before and collected after an additional 24 h of incubation (24 h + 24 h time point). To collect cells, the medium was discarded, the cells were washed 3 times with Phosphate Buffer Saline (PBS), and scraped from the bottom in 500 μL PBS. After a 5 min centrifugation at 500 × *g*, pelleted cells were solubilized in lysis buffer [150 mM NaCl, 50 mM Tris-HCl, pH 8.0, 5 mM ethylenediaminetetraacetic acid (EDTA), 2% (v/v) NP-40, 1× Phosphatase inhibitor (PhosStop, Roche), 1× Proteases inhibitors (complete Mini, Roche), 1 mM Phenylmethylsulfonyl fluoride (PMSF) and 0.1% (w/v) 1,4-Dithiothreitol (DTT)] for protein extraction, or in PBS for *GLUT1* and *GLUT3* expression evaluation performed as described above.

### Western Blot for GLUT1 and GLUT3 quantification

Pelleted cells solubilized in lysis buffer were incubated for at least 2 h, at −80 °C, and subjected to 5 cycles of 2 min 30 sec of ultrasounds with 30 sec intervals on ice between each cycle. Total extracts were then centrifuged (750 × *g*, 5 min) and supernatant was transferred to a new clean tube. The total amount of protein of each sample was quantified using Pierce 660 nm protein assay kit (Thermo Scientific) according to the manufacturer instructions, and 10 μg protein was used for the Western-Blot analysis. Protein extracts were first separated in a Sodium Dodecyl Sulfate polyacrylamide gel electrophoresis (SDS-PAGE) using a 10% (w/v) polyacrylamide gel with a 37.5:1 of acrylamide:bisacrylamide ratio (Merck Millipore) and then transferred onto a 0.45 μm PVDF membrane (GE Healthcare). After blocking membrane with 5% (w/v) low fat milk in TBST (50 mM Tris-HCl, pH 7.5, 150 mM NaCl, 0.1% (w/v) Tween-20), the blots were incubated for 1 h with a 1:200.000 dilution of GLUT1 antibody [EPR3915] (ab115730, Abcam) or with a 1:15.000 dilution of GLUT3 antibody [EPR10508(N)] (ab191071, Abcam), washed 3 times for 5 min with TBST, incubated another 1 h with 1:2.000 dilution of anti-IgG rabbit HRP-linked antibody (ref. 7074, Cell Signalling), washed again 3 times (5 min) with TBST and treated with WesternBright ECL (Advansta, USA) for signal acquisition in a Hyperfilm ECL (GE Healthcare). After stripping (0.1 M glycine, 20 mM magnesium acetate, 50 mM KCl, pH 2.2) and washing 3 times for 5 min with TBST, the membranes were blocked with 5% (w/v) low fat milk in TBST, incubated for 1 h with a 1:5.000 dilution of β-actin antibody (ref A5441, Sigma), washed 3 times for 5 min with TBST, incubated for 1 h with 1:3.000 dilution of anti-IgG mouse HRP-linked antibody (ref. 7076, Cell Signalling), washed 3 times for 5 min with TBST and treated with WesternBright ECL (Advansta, USA) for signal acquisition in a Hyperfilm ECL (GE Healthcare). The percentage of area of the protein band on each sample was measured using ImageJ2 software (Rueden *et al*., 2017).

The percentage of GLUT1 or GLUT3 in each sample was calculated by normalizing to the internal control (β-actin) and the corresponding control (cells treated with AuNP@PEG).

### Transport studies

Transepithelial transport experiments were performed using a similar procedure from the one described elsewhere^6^. Briefly, MKN-28 cells grown on polycarbonate transwell permeable inserts, 12 mm diameter, 0.4 µm pore size (Corning Costar, Corning, NY) at 75% confluence. Cells were cultured at 37 °C in an atmosphere of 5% CO_2_ and the medium was changed every two days. At 7 days MKN-28 cells were fully confluent and differentiated, so the transport experiments were initiated at that time. Experiments were conducted only in MKN-28 cell monolayers that showed a TEER > 150 Ω.cm^2^ (determined at 37 °C) measured using MILLICELL-ERS epithelial voltammeter (Millipore Co., Bedford, MA) with “chopstick” electrodes.

Before transport experiments and to further confirm that the addition of nanoconjugates did not affect cellular barrier integrity and cells toxicity, MKN-28 were seeded on 6.5 mm transwell inserts, 0.4 µm pore size, using 8 W TransFilter Adapter of ECIS system (Applied Biophysics, Troy, NY, USA). Cells were grown to confluence and transepithelial electrical resistance (TEER) was continually measured.

To further confirm the silencing efficacy of the nanoconjugates at the protein level in the transwell plates, cells were gently detached from the transwell membrane with PBS, centrifuged (500 × *g* for 5 min) and solubilized in lysis buffer for Western-Blot procedure, as described above.

After this treatment, media was removed and cells were washed with Hanks’ medium (HBSS), pH 7.4 at the basolateral side and with HBSS pH 5.0 at the apical side. Anthocyanin solution (500 µM) in HBSS pH 5.0 was added to the apical side of the cells and HBSS containing 2% fetal bovine serum free of polyphenols was added to the basolateral compartment. Transepithelial transport was followed as a function of time, at 4 °C and 37 °C. Samples were taken from the basolateral side and replaced by fresh medium. The samples (150 µL) were acidified with HCl 6 M to a final concentration of 1% and frozen at −18 °C until HPLC analysis.

### Molecular Docking

The crystal structures of human glucose transporters GLUT1 (PDBID: 4PYP, at 3.2 Å of resolution)^20^ and GLUT3 (PDBID: 4ZW9, at 1.5 Å of resolution^21^ were used to build the two systems. The GLUT1 transporter is in an inward-facing conformation and it is bound to a glucose analogue (*n*-nonyl-β-D-glucopyranoside (β-NG)), while GLUT3 is bound to D-glucose in an outward-occluded conformation. All crystallographic water and ligand molecules were removed, and polar hydrogen atoms were added considering the protonation state of the residues at physiological pH. The structures of the three anthocyanins (Pn3glc, Pn3HBsoph5glc and Pn3HBCsoph5glc) were build using the GaussView software^22^.

Ligand:protein docking calculations were performed with the VsLab software^23^ that uses the AutoDock 4.2 software^24^ to run the docking calculations and the VMD 1.9.2 program for visual inspection and analysis^25^.

The grid maps were centered into the glucose unit and comprised 54 × 54 × 70 points of 0.375 Å spacing. The Lamarckian genetic algorithm was employed (population size of individuals: 150; maximum number of energy evaluations: 2.5 × 10^6^; and maximum number of generations: 27000). For all the calculations, 50 docking rounds were performed with step sizes of 2.0 Å for translations and with orientations and torsions of 5.0°. Kollman partial charges were assigned to all transporters’ atoms. All docking conformations were clustered within 2.0 Å root-mean-square-deviations (RMSD) to prevent similar poses.

To validate the docking protocol, we employed our docking procedure for D-glucose, for which there was X-ray structural information considering both transporters (only the D-glucopyranoside atoms from the crystal GLUT1:sugar structure were considered). Considering the small RMSD values for the sugar heavy atoms, when comparing both the X-ray and top-ranked docking poses (2.4 Å and 2.6 Å, respectively), we deemed our docking procedure fit for describing the correct binding mode of D-glucose. Hence, they were used to evaluate the binding mode and affinities of the three phenolic compounds under evaluation. All docking poses were ranked by the binding energy values, and the protein-ligand complexes with the best energetic scores were taken as starting structures for the subsequent molecular dynamics (MD) simulations.

### Molecular Dynamics (MD) simulations

Based on energetic and structural criteria, the most favorable binding pose from the previous docking protocol, and considering each distinct transporter:ligand complexes, was incorporated into a POPC bilayer system, with 80 POPC lipids per layer. A hydration level close to 50 water molecules per lipid, and an ionic concentration of 0.15 M of NaCl was employed. Both membrane:GLUT-transporter models were constructed with the CHARMM-GUI webserver^26^.

The antechamber tool was used to parametrize the ligands, which were optimized with the HF/6–31 G(d) level of theory by using the Gaussian 09 software^27^. The RESP algorithm^28^ was used to calculate the atomic point charges to be employed in the MD simulations. The ff99SB^29^, GAFF^30^, and SLIPIDS^31^ force fields were used to characterize the proteins, the ligands and the membrane components, respectively. The TIP3P water model was employed. The starting geometry of each complex was minimized and equilibrated for 20 ns (with position restraints on protein atoms). Subsequently, the restraints were removed and each system was simulated for 100 ns. The SHAKE algorithm was applied to all bonds with hydrogen atoms^32^, which allowed for a 2 fs time-step using the Verlet leapfrog algorithm. Temperature and pressure were set to 310 K and 1 atm with the Langevin thermostat^33^ and the Berendsen barostat, respectively. Periodic boundary conditions were considered. A non-bonded cut-off value of 10 Å was used. Long-range electrostatic interactions were treated by a particle-mesh Ewald scheme^34^. All simulations were carried out using the PMEMD module, implemented in the AMBER 12.0 simulations package^35^. The results were analyzed with the CPPTRAJ tool^36^, and simulations were visually inspected with the VMD 1.9.2 program^25^.

The binding free energy values between the polyphenolic compounds and each GLUT transporter were determined using the Molecular Mechanics/Poisson Boltzmann Surface Area (MM/PBSA) approach^37^. 160 structures of each molecular complex were extracted over the last 80 ns of each MD simulation for analysis.

### Statistical analysis

All data was expressed as mean ± SEM from at least three independent experiments. Two-way ANOVA with Bonferroni’s multiple comparisons test and t-student test were performed using GraphPad Prism version 7.00 (GraphPad Software, San Diego California USA). Statistical significance was considered when p < 0.05.

## Results

### Effect of temperature in the transport of anthocyanins through MKN-28 gastric barrier

Natural anthocyanins are glycosylated flavonoids. In this study Malvidin-3-*O*-glucoside (Mv3glc) and Peonidin-3-O-glucoside (Pn3glc) have a glucose moiety at the C3 position and purple fleshed sweet potato (PFSP) anthocyanins have a glucose moiety at the C5 position and two additional glucoses at C3.

**Figure 1:**
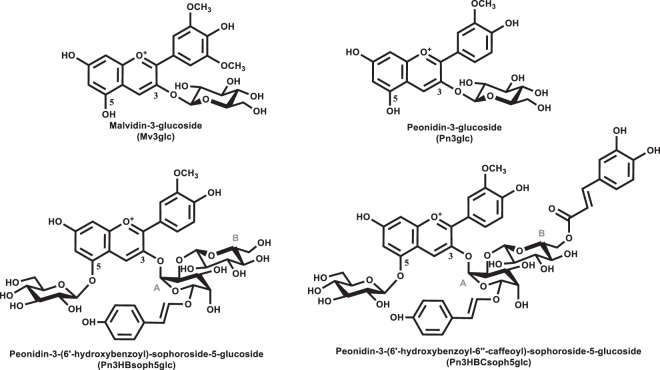
Chemical structures of the four anthocyanins assayed in the transepithelial transport studies.

The structures of the red wine anthocyanins Mv3glc and Pn3glc and the two main anthocyanins from PFSP Peonidin-3-(6′-hydroxybenzoyl)-sophoroside-5-glucoside (Pn3HBsoph5glc) and Peonidin-3-(6′-hydroxybenzoyl-6″-caffeoyl)-sophoroside-5-glucoside (Pn3HBCsoph5glc) are presented in Fig. 1.

In the case of anthocyanidins (anthocyanins lacking glucose) the hypothesis of transport by passive diffusion can be raised due to their higher hydrophobicity, but this hypothesis is not viable for anthocyanins due to the presence of the glucose moiety. It is highly unlikely for the glucose molecule to cross the cell membrane without a transporter system.

**Figure 2:**
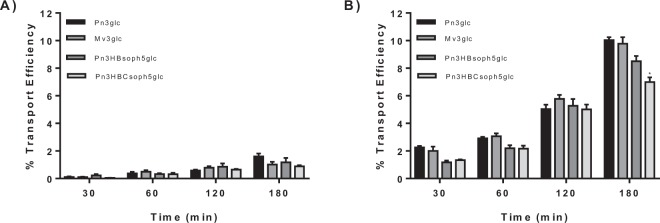
Transport efficiency of Pn3glc, Mv3glc, Pn3HBsoph5glc and Pn3HBCsoph5glc through MKN-28 barrier (Apical → Basolateral) at (**A**) 4 °C and (**B**) 37 °C. The experiments were conducted with apical pH 5.0 and basolateral pH 7.4. Results are presented as transport efficiency (%) (mean ± SEM). Transport efficiency percentages were calculated based on (compound concentrations at the basolateral side overtime)/(compound concentrations at the apical side at the zero hours)*100.

To demonstrate this, Mv3glc, Pn3glc and PFSP anthocyanins gastric transport was evaluated in MKN-28 cell barrier at 4 °C and 37 °C. The results obtained at 4 °C showed that the paracellular transport efficiency is below 2% (Fig. 2). Therefore, these results demonstrate that anthocyanins were actively transported by MKN-28 cells. Since glucose transporters had already been suggested as the main transporters involved in the absorption of these pigments^7^, the effect of their inhibition was further evaluated using a nano-based approach.

### Gold nanoparticle synthesis and characterization

Stable AuNPs with an average diameter of 14 ± 1 nm (Supplementary Fig. S1) were synthesized and functionalized with a PEG spacer for increased stability and biocompatibility^11,38^. These AuNP formulations represented the basic nanoparticle constructs for downstream functionalization with the respective antisense oligonucleotide sequences, targeting *GLUT1* and *GLUT3* mRNAs. In presence of the complementary sequence, the ssDNA hybridizes with the mRNA, forming a double stranded structure inhibiting the translation of mRNA into protein^39,40^.

The nanoconjugates were characterized by Transmission Electron Microscopy (TEM), UV-Vis spectroscopy, and Dynamic Light Scattering (DLS) (Supplementary Fig. S1). Data show an increase in the hydrodynamic radius after each step of functionalization with values of 19 nm for AuNPs, 21.5 nm for AuNP@PEG, and 31 nm for both AuNP@PEG@anti-*GLUT1* and AuNP@PEG@anti-*GLUT3*, indicating a successful functionalization. The antisense oligonucleotide coverage was determined by fluorescence-based assay with a final 1:40 and 1:30 AuNPs to oligonucleotide ratio for *GLUT1* and *GLUT3*, respectively.

### *GLUT1* and *GLUT3* gene silencing and effect at the protein level

**Figure 3:**
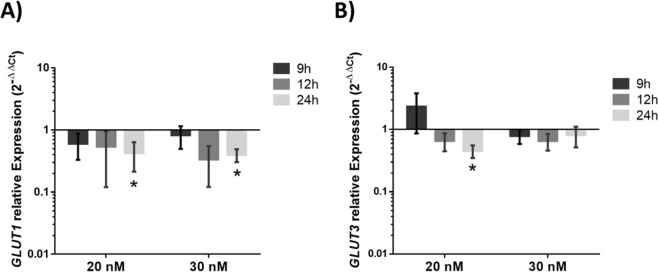
(**A**) *GLUT1* relative expression in MKN-28 cells incubated with 20 nM or 30 nM gold nanoparticles functionalized with 30% PEG and anti-*GLUT1* (AuNP@PEG@anti-*GLUT1*) for 9 h, 12 h and 24 h at 37 °C, 5% (v/v) CO_2_ and 99% (v/v) relative humidity. (**B**) *GLUT3* relative expression in MKN-28 cells incubated with 20 nM or 30 nM gold nanoparticles functionalized with 30% PEG and anti-*GLUT3* (AuNP@PEG@anti-*GLUT3*) for 9 h, 12 h and 24 h at 37 °C, 5% (v/v) CO_2_ and 99% (v/v) relative humidity. Gene expression variation is calculated via 2^−∆∆Ct^, using as reference *GAPDH* gene. Error bars represents SEM of at least three independent experiments. *p-value < 0.05 relative to relative expression at 9 h incubation of corresponding sample.

The most suitable conditions for *GLUT1* and *GLUT3* gene silencing (time and concentration of the silencing moieties) in the MKN-28 cell line were determined. A concentration of 30 nM of nanoconjugates induces a more noticeable down-regulation of *GLUT1*, while a concentration of 20 nM induces a higher down-regulation of *GLUT3* expression after 24 h of incubation (Fig. 3).

**Figure 4:**
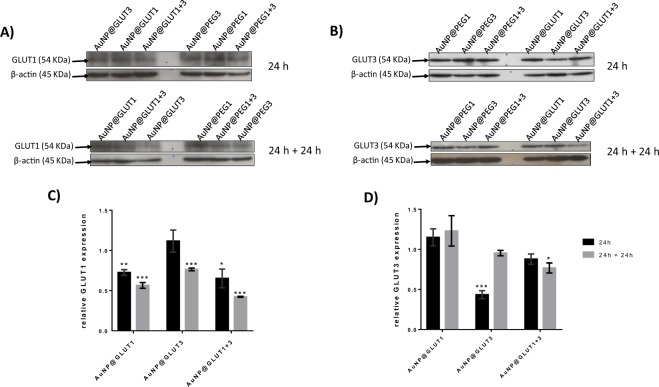
(**A**) Western Blot analysis of GLUT1 and β-actin proteins. (**B**) Western Blot analysis of GLUT3 and β-actin proteins. Represented Western Blots correspond to 10 µg total protein of MKN-28 cells grown on 24 well plates and incubated for 24 h with fresh RPMI medium supplemented with 5.5 mM fructose and 0.75 nM AuNP@PEG1, 0.63 nM AuNP@PEG3, 1.38 nM AuNP@PEG1 + 3, 30 nM AuNP@GLUT1, 20 nM AuNP@GLUT3, or a mixture of AuNP@GLUT1 + 3. After this 24 h period, cells were incubated for an additional 24 h with fresh medium supplemented according to the first incubation. (**C**) GLUT1 relative intensity values normalized to corresponding intensity of β-actin protein and to the corresponding AuNP@PEG control sample. (**D**) GLUT3 relative intensity values normalized to corresponding intensity of β-actin protein and to the corresponding AuNP@PEG control sample. Error bars represents SEM of at least three independent experiments. The grey line on the border of blots represent the place where images were cropped. Full length blot images can be found on Supplementary Fig. S2. *p-value < 0.5, **p-value < 0.005, ***p-value < 0.0005.

Afterwards, the silencing efficacy of the nanoconjugates under the conditions required for transport studies was evaluated at the mRNA and protein levels. Figure 4 shows a decrease in the expression of *GLUT1* and *GLUT3* genes in the presence of all nanoconjugates (AuNP@GLUT1, AuNP@GLUT3 or AuNP@GLUT1 + 3) and for both time points (24 h or 24 h + 24 h). However, the decrease of *GLUT1* expression was more pronounced in the presence of AuNP@GLUT1 and AuNP@GLUT1 + 3 nanoconjugates (compared to control (cells exposed to the same AuNP@PEG concentration)) (Fig. 4A). Concerning, *GLUT3* gene expression the decrease was more pronounced in MKN-28 cells incubated with AuNP@GLUT3 and AuNP@GLUT1 + 3 nanoconjugates (compared to control) (Fig. 4B). In this latter, the decrease of *GLUT3* gene expression was more pronounced after 24 h + 24 h incubation period (Fig. 4B).

**Figure 5:**
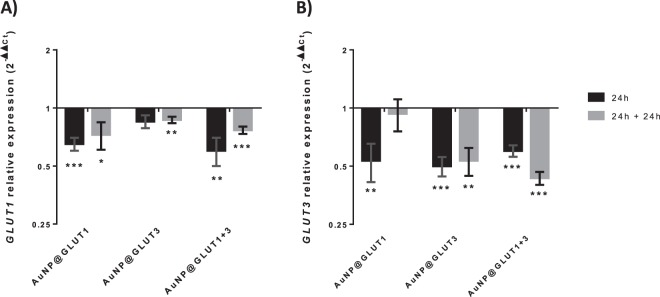
*GLUT1* (**A**) and *GLUT3* (**B**) relative expression in MKN-28 cells incubated for 24 h (black bars) or 24 h + 24 h (grey bars) with fresh RPMI medium supplemented with 5.5 mM fructose and 30 nM AuNP@GLUT1, 20 nM AuNP@GLUT3, or a mixture of AuNP@GLUT1 + 3. After 24 h cells were collected or incubated for an additional 24 h with fresh medium supplemented according to the first incubation. Gene expression was calculated through 2^−ΔΔCt^, using as internal reference *GAPDH* gene, and normalized to respective control samples consisting in MKN-28 cells treated with RPMI medium supplemented with 5.5 mM fructose and 0.75 nM AuNP@PEG (control of AuNP@GLUT1), 0.63 nM AuNP@PEG (control of AuNP@GLUT3), or 1.38 nM AuNP@PEG (control of AuNP@GLUT1 + 3), and collected at the same time point. Error bars represent the SEM of at least three independent experiments. *p-value < 0.5, **p-value < 0.005, ***p-value < 0.0005.

Expression of GLUT1 protein further demonstrate the silencing efficacy at the gene expression level, where a decrease of expression at 24 h + 24 h was also observed in cells incubated with AuNP@GLUT1 and AuNP@GLUT1 + 3 nanoconjugates (decrease of 0.4 and 0.6-fold, respectively, compared to control) (Fig. 5A, C). However, for the same incubation period, the amount of GLUT3 protein decreases slightly when cells were stimulated with the AuNP@GLUT1 + 3 nanoconjugate (Fig. 5B, D). The only exception to this trend was a higher reduction at the protein level observed for the first 24 h incubation period with the AuNP@GLUT3 nanoconjugate.

To understand whether the GLUT1 and GLUT3 protein expression variation pattern could be translated to assays using transwell plates, MKN-28 were cultured in transwell plates, submitted to the same treatment as described above and analysed after 24 h + 24 h incubation. The incubation period (24 h + 24 h) was selected based on the higher reduction of both gene and protein expression for GLUT1 and GLUT3.

**Figure 6:**
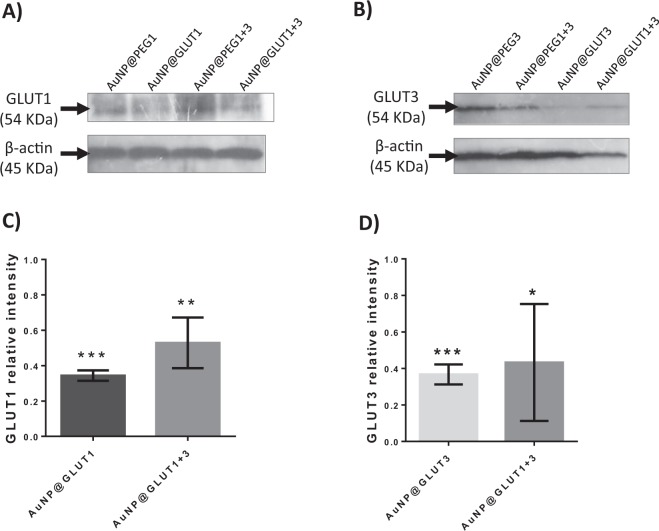
(**A**) Western Blot analysis of GLUT1 and β-actin proteins. (**B**) Western Blot analysis of GLUT3 and β-actin proteins. Represented Western Blots correspond to 10 μg total protein of MKN-28 cells grown on transwell plates and incubated for 24 h with fresh RPMI medium supplemented with 5.5 mM fructose and 0.75 nM AuNP@PEG1, 0.63 nM AuNP@PEG3, 1.38 nM AuNP@PEG1 + 3, 30 nM AuNP@GLUT1, 20 nM AuNP@GLUT3, or a mixture of AuNP@GLUT1 + 3. After this period of time, cells were incubated for an additional 24 h with fresh medium supplemented as previously. (**C**) GLUT1 relative intensity values normalized to corresponding intensity of β-actin protein and to the corresponding AuNP@PEG control sample. (**D**) GLUT3 relative intensity values normalized to corresponding intensity of β-actin protein and to the corresponding AuNP@PEG control sample. Error bars represents SEM of at least three independent experiments. The grey line on the border of blots represent the place where images were cropped. Full length blot images can be found on Supplementary Fig. S3. *p-value < 0.5, **p-value < 0.005, ***p-value < 0.0005.

Under these conditions, a more pronounced decrease of both GLUT1 and GLUT3 proteins expression is observed in all analysed samples, and a 70% decline of GLUT1 and GLUT3 expression in the presence of AuNP@GLUT1 and AuNP@GLUT3 nanoconjugates was observed (Fig. 6). The decreased abundance of these proteins is consistent with the inhibition of the *GLUT1* and *GLUT3* mRNA translation into proteins, suggesting an effective silencing mediated by AuNPs in the MKN-28 gastric barrier after 24 h + 24 h incubation and hence, further anthocyanins transport studies were performed in these conditions.

### Effect of glucose transporters inhibition on Mv3glc transport

Mv3glc is the main anthocyanin present in red wine. Due to the similarity in the transport efficiencies between Mv3glc and Pn3glc and since both have a glucose moiety at the same position the following assays were performed only with Mv3glc and PFSP anthocyanins.

No toxicity and no barrier impairment was observed after incubation of MKN-28 cells for 24 h + 24 h in the presence of the nanoconjugates (data not shown) which validates the nano-approach further applied.

**Figure 7:**
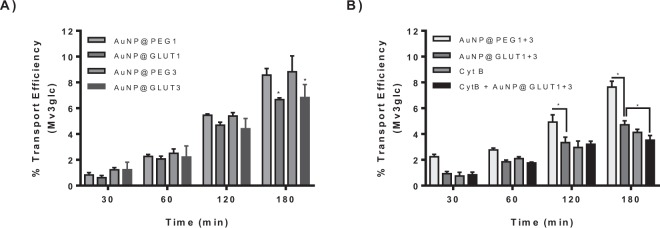
Transport efficiency of Mv3glc through MKN-28 barrier (Apical → Basolateral). The experiments were conducted with apical pH 5.0 and basolateral pH 7.4 in the presence of (**A**) 30 nM AuNP@GLUT1, 20 nM AuNP@GLUT3, or 0.75 nM AuNP@PEG (control of AuNP@GLUT1) or 0.63 nM AuNP@PEG (control of AuNP@GLUT3), (**B**) 1.38 nM AuNP@PEG (control of AuNP@GLUT1 + 3), a mixture of AuNP@GLUT1 + 3, Cythochalasin B (CytB, 50 µM) or a mixture of AuNP@GLUT1 + 3 and Cythochalasin B (50 µM). Significantly different from respective control *p < 0.05.

The transport of Mv3glc through the gastric cell barrier was evaluated after the incubation of MKN-28 cells for 24 h + 24 h in the presence of the nanoconjugates to induce the silencing of *GLUT1* and *GLUT3* genes. As observed in Fig. 7A it is possible to conclude that both GLUT1 and GLUT3 transporters are involved in the uptake of Mv3glc, since a relative reduction of transport around 25% was observed after 180 min in comparison with the respective control (AuNP@PEG). In addition, the inhibitory effect was much more pronounced and additive when both transporters were inhibited simultaneously (Fig. 7B), suggesting a synergistic effect of GLUT1 and GLUT3 in mediating Mv3glc transport. Previous studies showed that *GLUT1* and *GLUT3* isoforms are present in MKN-28 gastric cell line, being the latter the main one expressed^7^. The results highlight the implication of both isoforms, possibly due to the high homology degree between them.

The PFSP anthocyanins tested were transported through MKN-28 cell barrier with a kinetics that increased during incubation time, although the transport efficiency at 180 minutes (~8–7%) was lower than the one observed for Mv3glc (~10%). This difference may result from the steric hindrance effect conferred by the additional sugar moieties and the esterification with hydroxybenzoyl at C3 and with a caffeoyl group at glucose B.

**Figure 8:**
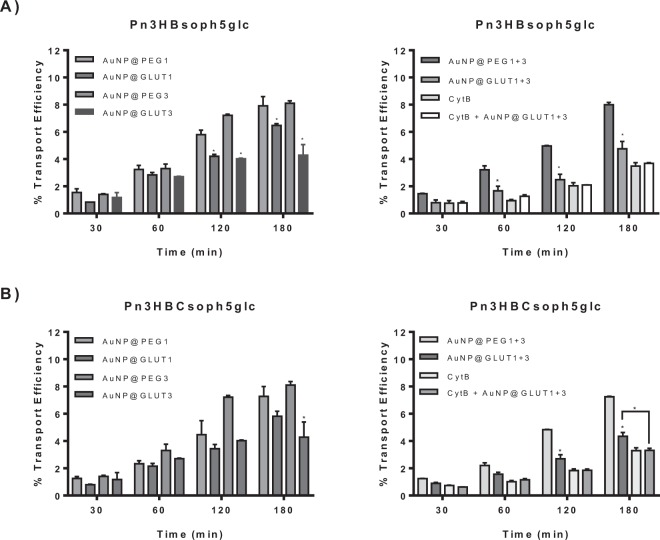
Transport efficiency of (**A**) Pn3HBsoph5glc and (**B**) Pn3HBCsoph5glc through MKN-28 barrier (Apical → Basolateral). The experiments were conducted with apical pH 5.0 and basolateral pH 7.4 in the presence of 30 nM AuNP@GLUT1, 20 nM AuNP@GLUT3, or 0.75 nM AuNP@PEG (control of AuNP@GLUT1) or 0.63 nM AuNP@PEG (control of AuNP@GLUT3), 1.38 nM AuNP@PEG (control of AuNP@GLUT1 + 3), a mixture of AuNP@GLUT1 + 3, Cythochalasin B (CytB, 50 µM) or a mixture of AuNP@GLUT1 + 3 and Cythochalasin B (50 µM). Significantly different from respective free oligonucleotide nanoparticle treatment * p < 0.05.

After treatment with AuNP@GLUT1 or AuNP@GLUT3, the two PSFP anthocyanins tested were less transported compared to the one observed for cells treated only with AuNP@PEG (Fig. 8), revealing a similar trend to the one previously observed for Mv3glc (Fig. 7). This reinforces the hypothesis of glucose implication in the absorption of these compounds, either at C3 or C5 position.

Since GLUT1 and GLUT3 are constitutively expressed at the cell apical membrane, part of the transport still observed after inhibition of *de novo* synthesis by treatment with AuNP@GLUT1 and AuNP@GLUT3 may result from the implication of the constitutive fraction. Due to the absence of total reversion of transport efficiency to the levels observed at 4 °C (below 2%) it was hypothesized that the constitutive glucose transporters could be the ones responsible for the transported anthocyanin to the basolateral side. This fact was confirmed by simultaneous addition of cytochalasin B and AuNP@GLUT1 + 3. Since the inhibitory effect was higher in the presence of cytochalasin B, the constitutive transporters seem to have an implication for the net transport (Figs 7 and 8). Even in the presence of cytochalasin B, total reversion to the paracellular levels was not accomplished. Therefore, the possible contribution of other transporters to the amount transported to the basolateral side was assessed using other specific inhibitors.

### Effect of organic transporters and efflux transporters inhibition

Additional studies were performed with Mv3glc using inhibitors of organic anion and cation transporters, since at pH 5.0 (pH of apical side) several anthocyanin forms may occur in equilibrium with other forms, including charged ones, such as quinoidal bases or flavylium cations^41^ and also with efflux inhibitors.

Probenecid, an inhibitor of transmembrane organic anion transporters (OAT1) with expression in MKN-28 cell line^42^ showed no effect on Mv3glc transport. In fact, at pH 5.0, a reduced amount of anionic species may be detected.

**Figure 9:**
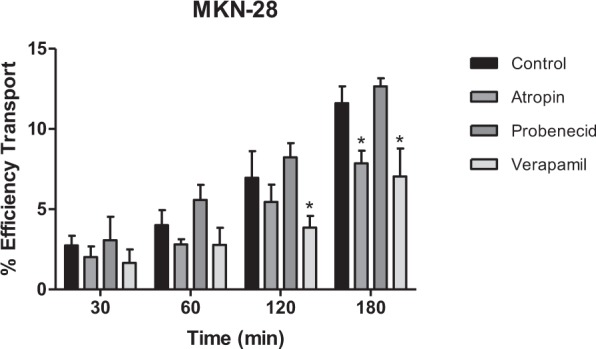
Transport efficiency of Mv3glc through MKN-28 barrier (Apical → Basolateral) in the presence of 50 µM of different inhibitors. The experiments were conducted with apical pH 5.0 and basolateral pH 7.4. Significantly different from control (AuNP@PEG) for the same incubation time *p < 0.05.

Atropin, an inhibitor of organic cation transporter (OCT1) and verapamil known as a P-glycoprotein inhibitor, both expressed in MKN-28 cells^43,44^, were able to reduce the amount of Mv3glc detected in the basolateral side of the gastric barrier model. The first one may be related to the decrease of absorption of Mv3glc in the flavylium cation form and the latter with the inhibition of efflux transporters. As a consequence of efflux transporters inhibition, a cytosolic accumulation of Mv3glc may be indirectly inferred by the decrease in the amount observed in the basolateral side (Fig. 9).

### Molecular Docking and Molecular Dynamics (MD) simulations

Due to the differences observed in the transport efficiency of the two PFSP anthocyanins compared with Mv3glc different computational simulations were used to assess the implication of their complexity degree.

To evaluate the interaction of the molecules Pn3glc (precursor), Pn3HBsoph5glc and Pn3HBCsoph5glc in hemiketal form (main form at pH 5.0) against both GLUT1 and GLUT3 transporters, molecular docking allied to MD simulations was performed. The energies and the binding mode of compounds Pn3HBsoph5glc and Pn3HBCsoph5glc are quite similar between the top-two docking solutions (data not shown). Considering the little difference of the binding free energy of the two docking solutions for all compounds (maximum difference of 1.4 kcal.mol^−1^), only the first docking solutions were chosen to perform the MD simulations.

**Figure 10:**
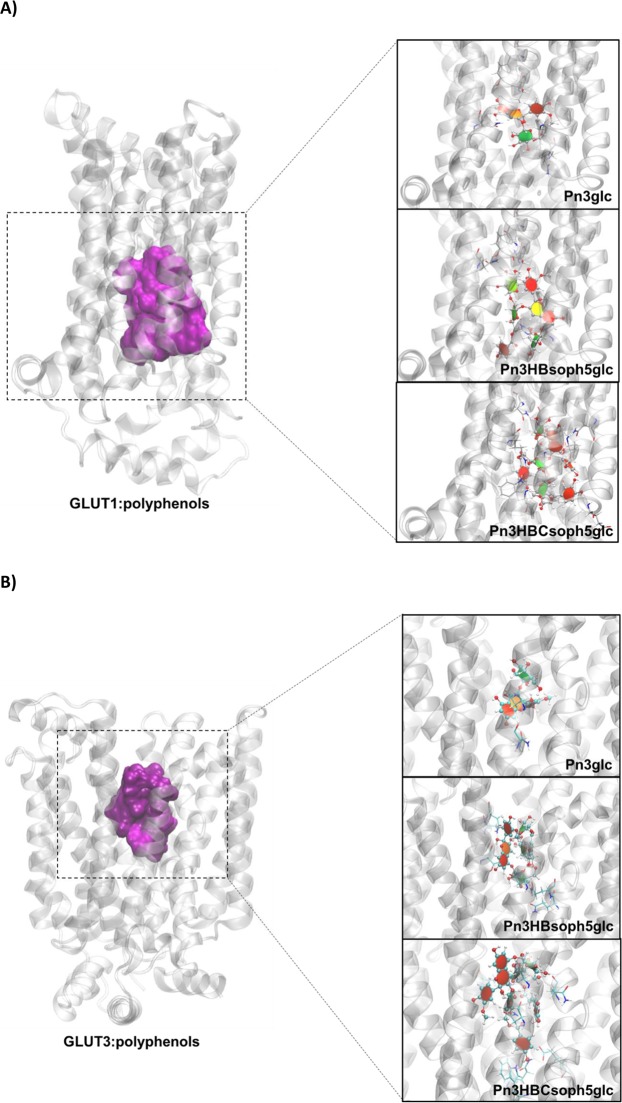
Representation of the optimized structure of each (**A**) GLUT1:polyphenol complex and (**B**) GLUT3:polyphenol complex. The main amino acids involved in the polyphenols’ binding are also shown (depicted with sticks and colored by atom type). GLUTs are represented in cartoon and colored in gray, while the polyphenol compounds are represented as ball-and-sticks and colored by atom type. Aromatic, non-aromatic saccharide, and non-aromatic non-saccharide rings of each polyphenol are colored in red, green and orange, respectively. The binding region of GLUTs for all polyphenols is represented as a purple surface.

Figure 10A,B show the binding region of each GLUT1:polyphenol and GLUT3:polyphenol complex, respectively. It was observed that the polyphenols’ moieties that interact more deeply within both GLUT channels are the glucose and AC rings. This suggests that these groups may have a key role in the binding and translocation of these ligands through the two transporters. Interestingly, it was verified that the larger compounds (PFSP anthocyanins) showed frequent/stable intramolecular interactions, in which the phenolic rings establish π-π and CH- π stacking contacts. These results suggest that the larger polyphenols should present a similar translocation mechanism, through the free glucose moieties. This agrees with the previous proposal for the anthocyanins’ crossing mechanism through human GLUT receptors^7^.

The analysis of the MD trajectories revealed that all anthocyanins remained bound to the same GLUT region throughout the entire simulations, suggesting the establishment of stable intermolecular interactions. In all simulations, the major moieties of polyphenols involved in H-bonds seems to be glucose and AC rings that may highlight their important role in the polyphenols transmembrane transport (data not shown).

## Discussion

It is generally acknowledged that anthocyanins are beneficial to human health, although the mechanisms of their absorption are not yet fully described. The long-term view that anthocyanins could only be absorbed in their aglycon forms has been changing, since anthocyanins are unique compared with other flavonoids once that they may be absorbed as intact glycosylated forms. Native anthocyanins have been detected in both plasma and urine of human subjects few minutes after oral consumption of blackberry juice or red wine^4,5^. Candidates for anthocyanin transporters are glucose transporters since anthocyanins possess a sugar moiety, which consists in most cases in glucose residues.

Recently, Zou and coworkers reported that Cy3glc intestinal absorption decreased after *SGLT1* or *GLUT2* siRNA transfection^45^. More recently, a study with rats showed that although the overall uptake of anthocyanins was not affected by the co-administration of glucose, the kinetics of the uptake was suggesting the involvement of glucose transporters. It was also found a significant correlation between SLGT1, GLUT2 and anthocyanin absorption through computational studies^46^. Furthermore, Zhang and coworkers demonstrated the bioaccessibility and bioavailability of anthocyanins from purple roots, suggesting, once again the involvement of SLGT1 and GLUT2 as the main candidates on anthocyanins transport at the intestinal level^47^.

Over the years, attention has been given to intestinal anthocyanin uptake as the main site of absorption in the body. However, the findings of anthocyanins in the bloodstream as early as 15 minutes are not compatible with an exclusive intestinal uptake.

Several studies using a human gastric model have pointed towards the possible involvement of glucose transporters in the transport of anthocyanins^6,7^. However, the direct involvement of such transporters has not been clearly demonstrated since only indirect approaches were used to assess this matter. So, the need to have a molecular approach targeted to specific proteins/transporters becomes evident.

In this work a nano-based approach was used to clearly associate the absorption of anthocyanins with glucose transporters by silencing *GLUT1*, *GLUT3* or both.

The structurally related anthocyanins selected intended to support the importance of glucose position and substituents to the transporter binding. The hindrance effect of the sugar moieties and the substituents on PFSP anthocyanins (~8–7%) are clear when compared to the native anthocyanin Pn3glc (~10%), demonstrated by the reduction of the transport efficiency of PSFP anthocyanins at the higher incubation time.

Also, it is interesting to notice that besides this, the results for the samples treated with nanoparticles with antisense for *GLUT1* or *GLUT3* showed similar trends for all the anthocyanins tested, suggesting that the position of the free glucose residue is not an important feature.

The computational results suggest that all anthocyanins are able to bind to both human GLUT1 and GLUT3 transporters, with the bigger compound Pn3HBCsoph5glc showing the highest affinity of the three evaluated compounds, since it is the one that establishes the highest number of H-bonds with the transporters. The AC rings and the glucose unit (glc) seem to have the most important role in the anthocyanin transmembrane binding. These groups establish both hydrophilic (H-bonds) and hydrophobic (CH-π and π-π stacking) interactions that are crucial for the anthocyanin binding into the human glucose transporters.

Although a clear reduction in anthocyanin transport was observed upon gold nanoparticles treatments, only when a 4 °C treatment was applied a total reduction of active transport was observed. This is suggested by the different values for treatments with gold nanoparticles and 4 °C treatment. So, relative differences of about 38 up to 41% depending on the cases when comparing AuNP@PEG with or without antisense *GLUT1/3*, suggest the involvement of other transporters. The treatments with inhibitors of different transporter families showed a reduced absorption of Mv3glc, although not so significant as in the case of GLUTs inhibition. This phenomenon is quite understandable since cells have a high number of mechanisms to internalize and transport different compounds, and it is virtually impossible to assess all of them to be sure of which of them are involved. In fact, Passamonti and coworkers demonstrated the involvement of bilitranslocase on anthocyanins uptake at gastric level^48^.

Anthocyanins display some chemical unique features such as their dependence on pH^49^. The fact that these compounds can reach an equilibrium in different structural forms at a specific pH value, suggest that anthocyanin transport *in vivo* is dynamic, and cannot be attributed to a specific form. At pH 5, the working pH of the model used in this work and the average pH of the stomach at the fed state, anthocyanins are found at different forms in equilibrium^41^,^49^. This may explain the results obtained when testing different transporter families, which highlighted that this particular class of polyphenols can be transported in different equilibrium forms by different transporters.

## Conclusion

This study provides useful data to assist the clarification of the role of glucose transporters in the gastric absorption of anthocyanins. This data also suggest the involvement of other type of transporters that may contribute to the global transport efficiency, which should be the focus of future studies into this theme. This work brings new insights onto the paradox between the high levels of anthocyanins consumed, their low bioavailability and the reported health effects.

## Supplementary information

Supplementary info file
