# Supplementary material for: GLUT1 and GLUT3 involvement in anthocyanin gastric transport- Nanobased targeted approach
Source: Sci Rep. 2019 Jan 28;9:789. doi: 10.1038/s41598-018-37283-2 (PMC6349854; doi:10.1038/s41598-018-37283-2)
Supplement: Supplementary file 1 — Supplementary info file [file 41598_2018_37283_MOESM1_ESM.pdf]

Supplementary Information

GLUT1 and GLUT3 involvement in anthocyanin gastric transport - Nanobased targeted approach.

Hélder Oliveira[1, ¥], Catarina Roma-Rodrigues[2, ¥], Ana Santos[2], Bruno Veigas[2], Natércia Brás[3], Ana Faria[1,4,5,6], Conceição Calhau[4,5,6], Victor de Freitas[1], Pedro V. Baptista[2,*], Nuno Mateus[1], Alexandra R. Fernandes[2], Iva Fernandes[1,*]

[1]REQUIMTE/LAQV, Department of Chemistry and Biochemistry, Faculty of Sciences, University of Porto, 4169-007 Porto; Portugal

[2]UCIBIO, Departamento de Ciências da Vida, Faculdade de Ciências e Tecnologia, Universidade Nova de Lisboa, Campus de Caparica, 2829-516 Caparica, Portugal;

[3]REQUIMTE/UCIBIO, Department of Chemistry and Biochemistry, Faculty of Sciences, University of Porto, 4169-007 Porto; Portugal

[4]Nutrition & Metabolism, NOVA Medical School|Faculdade Ciências Médicas Universidade Nova de Lisboa, 1169-056 Lisboa, Portugal

[5]CINTESIS, Center for Health Technology and Services Research, Porto, Portugal

[6]Comprehensive Health Research Centre, NOVA Medical School|Faculdade Ciências Médicas, Universidade Nova de Lisboa, 1169-056 Lisboa, Portugal

¥ both authors contributed equally

Corresponding author. Iva Fernandes and Pedro V Baptista, *e-mail: iva.fernandes@fc.up.pt, telephone: +351.220402562 and pmvb@fct.unl.pt, telephone: +351212948530.

Figures

	TEM (nm)	SPR Peak	DLS size(nm)
AuNPs	14 ± 1 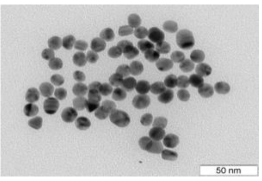	520	19
AuNP@PEG		521	21.5
AuNP@PEG@anti-*GLUT1*		524	31
AuNP@PEG@anti-*GLUT3*		524	31

Figure S1 – Characterization of gold nanoconjugates. Gold nanoparticles (AuNPs), AuNPs functionalized with 30 % PEG (AuNP@PEG) and AuNP@PEG functionalized with anti-GLUT1 (AuNP@PEG@anti-GLUT1) or anti-GLUT3 (AuNP@PEG@anti-GLUT3) through Transmission Electron Microscopy (TEM), Dynamic Light Scattering (DLS) and UV-Vis (Surface Plasmon Resonance, SPR peak).

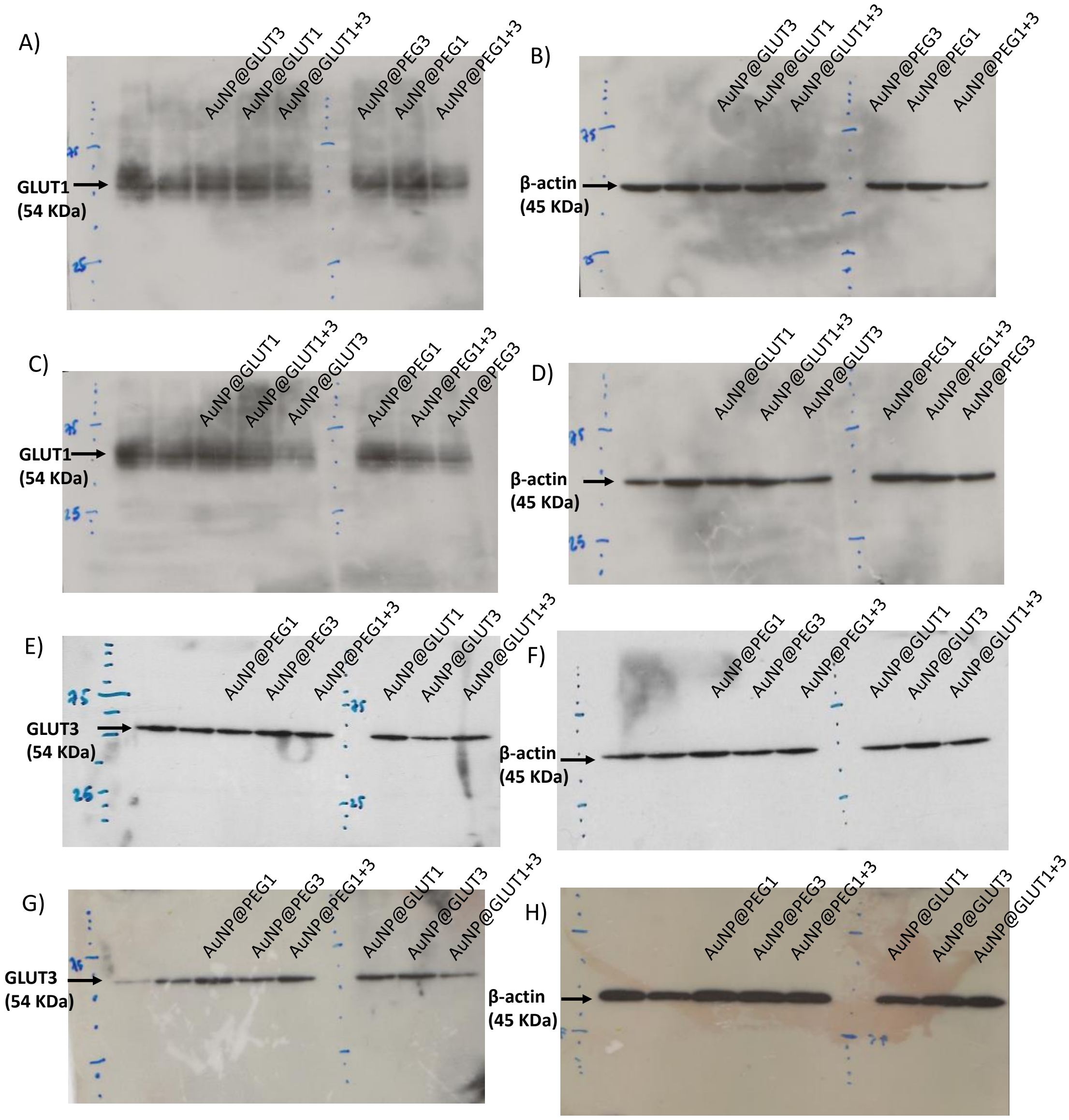

Figure S2 – Full length blots of the Western Blot analysis of A) GLUT1 after 24 h; B) β-actin, control of GLUT1 blot after 24 h; C) GLUT1 after 24h+24h and D) β-actin, control of GLUT1 blot after 24h+24h. E) GLUT3 after 24 h; B) β-actin, control of GLUT3 blot after 24 h; C) GLUT3 after 24h+24h and D) β-actin, control of GLUT3 blot after 24h+24h. Represented Western Blots correspond to 10 µg total protein of MKN-28 cells incubated for 24 h or 24 h plus 24 h with fresh RPMI medium supplemented with 5.5 mM fructose and 30 nM AuNP@PEG@anti-*GLUT1* (AuNP@GLUT1), 20 nM AuNP@PEG@anti-*GLUT3* (AuNP@GLUT3), or a mixture of 30 nM AuNP@PEG@anti-*GLUT1* and 20 nM AuNP@PEG@anti-*GLUT3* (AuNP@GLUT1+3). After 24 h cells were collected or incubated for an additional 24 h with fresh medium supplemented according to the first incubation. Control samples consist in MKN-28 cells treated with RPMI medium supplemented with 5.5 mM

fructose and 0.75 nM AuNP@PEG (control of AuNP@GLUT1), 0.63 nM AuNP@PEG (control of AuNP@GLUT3), or 1.38 nM AuNP@PEG (control of AuNP@GLUT1+3), and collected at the same time point.

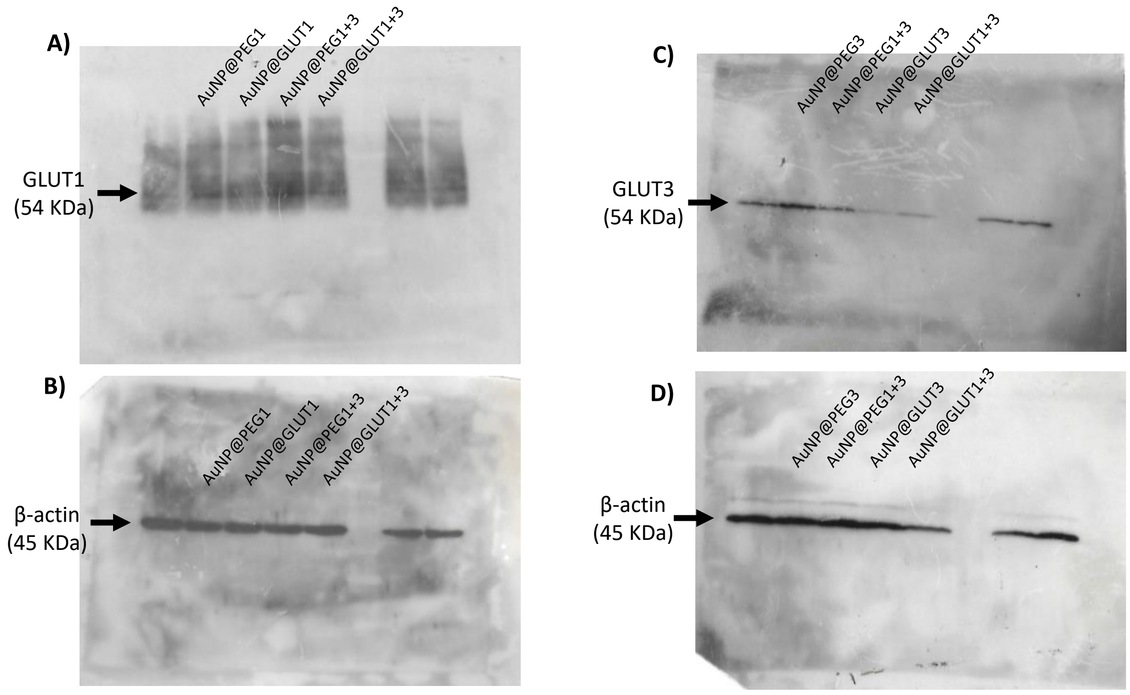

Figure S3 – Full length blots of the Western Blot analysis of A) GLUT1; B) β-actin, control of GLUT1 blot; C) GLUT3 and D) β-actin, control of GLUT3 blot. Represented Western Blots correspond to 10 µg total protein of MKN-28 cells grown on transwell plates and incubated for 24 h with fresh RPMI medium supplemented with 5.5 mM fructose and 0.75 nM AuNP@PEG (AuNP@PEG1), 0.63 nM AuNP@PEG (AuNP@PEG3), 1.38 nM AuNP@PEG (AuNP@PEG1+3), 30 nM AuNP@PEG@anti-GLUT1 (AuNP@GLUT1), 20 nM AuNP@PEG@anti-GLUT3 (AuNP@GLUT3), or a mixture of 30 nM AuNP@PEG@anti-GLUT1 and 20 nM AuNP@PEG@anti-GLUT3 (AuNP@GLUT1+3). After 24 h cells incubated for an additional 24 h with fresh medium supplemented according to the first incubation.
